# Testicular pathology, gonadal and epididymal sperm reserves of Yankasa rams infected with experimental *Trypanosoma brucei brucei* and *Trypanosoma evansi*

**DOI:** 10.14202/vetworld.2016.759-765

**Published:** 2016-07-23

**Authors:** Yunusa A. Wada, Sonnie J. Oniye, Peter I. Rekwot, Oluyinka O. Okubanjo

**Affiliations:** 1 Department of Biological Sciences, Faculty of Science, Ahmadu Bello University, Zaria, Nigeria; 2 National Animal Production Research Institute, Ahmadu Bello University, P.M.B. 1096 Shika, Zaria, Nigeria; 3 Department of Veterinary Parasitology and Entomology, Faculty of Veterinary Medicine, Ahmadu Bello University, Zaria, Nigeria

**Keywords:** gonadal sperm reserve, mixed infection, testicular degeneration, *Trypanosoma brucei brucei*, *Trypanosoma evansi*, Yankasa ram

## Abstract

**Aim:**

The study was conducted to evaluate the pathological effects of trypanosomosis on the testes, gonadal, and epididymal sperm reserves of Yankasa rams for 98 days.

**Materials and Methods:**

A total of 16 Yankasa rams, aged between 24 and 30 months and weighed between 22 and 25 kg, were acclimatized for a period of 2-months in a clean fly proof house and were adequately fed and given water *ad-libitum*. Of the 16 rams, 12 that were clinically fit for the experiment at the end of the acclimatization period were randomly divided into four groups: Groups I, II, III, and IV, each having 3 rams. Groups I and II were each challenged singly with experimental *Trypanosoma brucei brucei* (Federer strain) and *Trypanosoma evansi* (Sokoto strain), respectively, while Group III was challenged with mixed *T. brucei brucei* and *T. evansi* parasites (50% of each species in the infective inoculum) and Group IV was left as an uninfected control. Each infected ram received 2 mL of the infected blood containing 2×10^6^ trypomastigotes via the jugular vein, while the control group received 2 mL each, normal saline.

**Results:**

All the infected rams developed clinical signs typical of trypanosomosis at varying pre-patent periods. The gross lesions observed in the infected rams in Group II were moderate and more severe in those of Groups I and III. Histological sections of the testes of infected rams (Groups I, II, and III) showed moderate (*T. evansi*-infected group) to severe (mixed and *T. brucei brucei*-infected groups) testicular degenerations with reduction in number of spermatogenic cell layers, degenerated seminiferous tubules, congested interlobular spaces, loss of tissue architecture with significant (p<0.01) depletion, and loss of gonadal and epididymal sperm reserves in Groups I and III in comparison to Group II and the control Group IV. No observable clinical signs and histopathological lesions were found in those rams of the control Group IV.

**Conclusion:**

The study concluded that trypanosomosis due to experimental *T. brucei brucei* or *T. evansi* or mixed infections (of both parasites) caused testicular damage, decreased epididymal and gonadal sperm reserves and an important cause of infertility in Yankasa rams.

## Introduction

Pathogenic animal trypanosomes are causative agents of the most common livestock diseases which have an important economic impact on many African countries. These diseases usually cause debilitating symptoms manifested by anemia and cachexia which may result in death. In Nigeria, demand for ram is on the increase due to its importance in religious and naming ceremonies, and its culinary attraction at barbeque stands; but parasites, especially trypanosomes, have become a major threat to sustainable livestock production resulting in a decline in production [1]. Apart from hematological and pathogenic aberrations that do occur in animals infected with the parasites, recent studies show that they cause a wide range of reproductive disorders in animals, including degeneration of the hypothalamus, pituitary glands, and gonads with consequent disruptions in the secretions and plasma concentrations of the hormones necessary for normal reproductive processes in both sexes [2,3].

In female animals, the effect includes severe genital lesions, temporary or permanent anestrus, and abnormal estrous cycles. In addition, trypanosomal-induced death during pregnancy, abnormal pregnancy, dystocia, abortion, premature birth, low birth weight, stillbirth, transplacental fetal infection, neonatal death, and other pathogenic effects on fetuses and offspring have been reported by Batista *et al*. [2] and Silva *et al*. [3].

In the males, the effects include delayed puberty, loss of libido, severe degenerative changes of the genitalia, manifested by the production of very poor quality semen, and loss of epididymal and gonadal sperm reserves [4,5]. Testicular atrophy has also been reported with the presence of dead spermatozoa in the seminiferous tubules of goats [4,6]. Infection in rabbits with *Trypanosoma brucei brucei* causes increased scrotal circumference, scrotal inflammation, severe testicular degeneration, abnormal spermatogenesis, aspermatogenesis, and incomplete resolution of the genital organs [7]. The presence of *T. brucei brucei* in the genital tract and the brain, in addition to severe lesions resulting in the degeneration of testes which involved the Leydig cells, basement membrane, Sertoli and germ cells, with resultant loss of libido was reported in boars [8]. Animals that survived infection often remained infertile [9]. In *Trypanosoma vivax* or *Trypanosoma congolense* infected Yankasa rams, the effects on reproduction ranges from scrotal edema, poor semen quality or the cessation of semen production, genital lesions, degeneration of the testes, loss of libido as well as infertility and loss of epididymal and gonadal sperm reserves [4,5,10].

Considering the fact that sheep are often graze alongside cattle or camels from northern Nigeria toward the southern vegetation belts by herdsmen in search of greener pastures, there is the possibility of exposing them to the risk of bites by tsetse flies and other stomoses, which are invertebrate hosts of trypanosomes. This study was, therefore, designed to determine the extent of *T. brucei brucei* and *T. evansi* and mixed infections on the testes, epididymal, and gonadal sperm reserves of Yankasa rams.

## Materials and Methods

### Ethical approval

The experimental protocols and treatments were done in accordance with the principles of Animal Use and Care (ABUCAUC) of Ahmadu Bello University, Zaria.

### Experimental animals

A total of 16 apparently healthy and intact Yankasa rams, aged between 24 and 30 months and weighed between 22 and 25 kg, were purchased from Sheme market, an apparently tsetse-free zone, in Katsina State of the Nigerian Sudan-Guinea Savannah. The ages of the animals obtained from the market were confirmed by the presence of temporary and permanent incisors as described by Wilson and Durkin [11], that is, by the eruption of permanent incisors at permanent corners of the cheek.

### Housing and screening of experimental animals

The purchased animals were housed in an insect-proof animal pen at the Department of Veterinary Parasitology and Entomology, Faculty of Veterinary Medicine, Ahmadu Bello University, Zaria, where they were screened for the presence of ectoparasites, endoparasites, and hemoparasites. The rams were thereafter treated with Oxytetracycline (Tridax^®^) intramuscularly, at a dose of 20 mg/kg body weight and Albendazole (Sambezole^®^, Sam Pharmaceutical Ltd; Animal Care, Nig. Ltd.) orally, at a dose of 7.5 mg/kg body weight. The rams were sprayed against ectoparasites with Diazinon (Diaznol^®^, Animal Care, Nig. Ltd.), at concentration of 2 mL/L of water. They were acclimatized for 8 weeks and neck tagged for the purpose of identification.

### Acclimatization and physical examination of animals

The rams were fed with wheat offal, ground-nut and cowpea hays, fresh grasses (whenever available), and salt licks. Water was supplied *ad*-*libitum*. During the 8 weeks acclimatization period, they were subjected to routine handlings such as physical examination, determination of the body weight, rectal temperature, scrotal circumference, semen collection, and collection of blood samples for screening of hemoparasites and determination of baseline semen and hematological indices. Before commencement of the experiment, the rams were ensured to be clinically free of trypanosomes and other hemoparasites in their blood using buffy coat centrifugation technique [12,13].

#### Source of trypanosomes

*T. evansi* was obtained from an infected camel at slaughter in Sokoto abattoir, Sokoto State, North-Western Nigeria, while *T. brucei brucei* was obtained from the Nigerian Institute for Trypanosomiasis Research, Kaduna, Nigeria, originally isolated from a natural infection in cattle, in Kaduna State. Both parasites were maintained in Wistar rats by serial passage and were transported to the Protozoology Laboratory, Department of Veterinary Parasitology and Entomology, Faculty of Veterinary Medicine, Ahmadu Bello University, Zaria, for proper identification using the Giemsa-stained thin blood smear diagnostic technique [14]. *T. evansi* was identified morphologically by the characteristic free and long flagellum with well-developed undulating membrane, subterminal kinetoplast, and an elongated and centrally placed nucleus while *T. brucei brucei* was identified by the presence of short posterior kinetoplast, long and conspicuous undulating membrane with no free flagellum. Both parasites were sub-inoculated intraperitoneally into 10 Wistar rats each and were kept in separate cages. The rats were fed with commercial pelleted feed and water supplied *ad-libitum*. Before inoculation into experimental rams, blood samples were collected daily from each of the rats to determine the level of parasitemia using the hematocrit centrifugation technique (HCT) as described by Woo [12] and Biryomumaisho *et al*. [13].

### Experimental design

By the end of the 8 weeks acclimatization period, 4 of the 16 rams that were deemed unfit (due to poor semen characteristics) for the experiment were eliminated. The remaining 12 that were clinically fit (due to good semen characteristics and higher hematological values) for the experiment were randomized into four experimental groups (I, II, III, and IV) of three rams each, based on their mean packed cell volumes (PCV) and weights. The rams in Groups I, II, and III were experimentally infected with *T. brucei brucei*, *T. evansi*, and mixed inoculum of both parasites, respectively, while those in Group IV served as the uninfected control.

### Inoculation of experimental animals

After detection of the trypanosome parasites in the blood of the inoculated rats, they were monitored to their peak value (30-40 in buffy coat layer per field). All the infected rats became parasitemia within 3-14 days post inoculation. The rats were bled using sterilized surgical blades through the cardiac (heart) puncture to collect sufficient blood into Bijou bottles, containing 2 mg of ethylene diamine tetraacetic acid for the inoculation of the rams in Groups I, II, and III. The dosage of *T. evansi* and *T. brucei brucei* used for inoculation was estimated using the rapid matching wet-examination technique described by Herbert and Lumsden [15]. A drop of mouse blood was examined under the 40× magnification of a microscope, the number of trypanosomes in each field counted and matched with log figure obtained from a reference table [15].

Group I: Each ram was inoculated via the jugular vein with 2 mL of blood containing 2×10^6^ *T. brucei brucei*.

Group II: Each ram was inoculated via the jugular vein with 2 mL of blood containing 2×10^6^ *T. evansi*.

Group III: Each ram was inoculated via the jugular vein with 2 mL of blood containing 1×10^6^ *T. evansi* and 1×10^6^ *T. brucei brucei*.

Group IV: Served as the uninfected control, each ram received 2 mL normal saline.

### Observation of clinical signs

Clinical signs that were investigated during the study include rise in rectal temperature, weight gain, scrotal diameter, reduced feed intake, loss of body condition, weakness, dullness, roughy hair coat, PCV, hemoglobin concentration, total plasma protein, and anemia.

### Determination of gonadal sperm/spermatid reserves

At the end of the experiment (day 98 post infection [p.i.]), a total of 8 rams (two from each group) were randomly selected from the experimental rams. They were slaughtered to determine their gonadal sperm/spermatid reserves; that is, their integrity to support gonadal functions. The two testes of each of the 8 rams were carefully removed and labeled for proper identification. A small portion of the testes from each of the sacrificed ram was removed and preserved in Bouin solution for histopathological examinations. The weight, length, and volume of each testis were determined. Gonadal sperm/spermatid reserves were determined according to the methods described by Rekwot *et al*. [16] and Ogunlade *et al*. [17]. Briefly, the tunica albuginea of each testis was removed using a scalpel blade to expose the testicular parenchyma which was weighed, sliced, and homogenized with a high speed blender for 2 min with 50 mL of 0.9% sodium chloride (NaCl) containing antibiotics (sodium penicillin G, 100 IU/mL and streptomycin sulfate, 1 mg/mL) to prevent bacterial growth. The homogenate volume, after rinsing the blender container with 20 mL of saline was measured. About 5 mL of the homogenate was transferred to a conical flask and further dilution was made with 40 mL of saline, and the homogenate was stored overnight at 5°C to allow sperm cells to ooze out of the tissues. Finally, the gonadal sperm/spermatid concentration was determined with a hemocytometer according to the method of Coles [18]. The concentration of spermatozoa was determined using the erythrocyte counting chamber of the hemocytometer that was crossed with microscopic grids containing small squares. Sperm cells and spermatids were counted diagonally from top left to bottom right in five large squares as described by Rekwot *et al*. [16].

### Determination of epididymal sperm reserves

In determining the epididymal sperm reserves, the epididymis was carefully separated from the testis with a scalpel and the lengths and weights of the caput (head), corpus (body), and cauda (tail) epididymis were determined, minced separately into 20 mL of 0.9 % NaCl solution with a pair of sharp scissors, and the homogenates were stored overnight at 5°C to allow sperm cells to ooze out of the tissues. The samples were thereafter, filtered through gauze and the filtrate volume was measured and recorded. 1 mL each of epididymal portion was diluted separately with 2 mL of saline solution. Finally, the concentration of sperm reserve was determined using the hemocytometer and light microscopy [16,17].

### Pathological studies

At the end of the experiment, the selected 8 rams were sacrificed and subjected to postmortem examinations, and biopsies were taken for histopathology. Tissue samples collected from the testes were preserved in Bouin solution. The Bouin fluid mixture was prepared by mixing 75 mL of picric acid saturated aqueous solution (2.1%), 25 mL of 40% formaldehyde, and 5 mL of glacial acetic acid. After 48 h of fixation, the tissue samples were processed (washed in 50% and 70% alcohol), embedded in paraffin wax, and sectioned at 3-5 µ. The sections were mounted on clean grease-free glass slides and stained with hematoxylin and eosin stains as described by Luna *et al*. [19]. The stained slides were examined microscopically at 40× objective. Histopathological lesions were observed, recorded, and photomicrographed with the aid of a digital camera (Cannon, 16 Mpx). Sections from the testes were examined, and the degrees of testicular degeneration (Table-1) were scored as mild, moderate, or severe as described by Sekoni *et al*. [20].

**Table-1:** Grading of genital degeneration in ram infected with *Trypanosoma* spp.

Grade	Testes	Epididymis
Severe (+++)	Germinal epithelial layers reduced to one or two with predominantly Sertoli cells. 60% plus tubules affected	Lesions observed in 60% or more tubules
Moderate (++)	Germinal epithelial layers reduced to 3 with at least 50% of tubules affected	Lesions observed in at least 50% of tubules
Mild (+)	Most tubules normal with slight reduction in the Germinal layers	Most tubules are normal

### Data analysis

The data obtained were analyzed using Microsoft Office Excel, 2010. Intergroup comparisons of mean gonadal and epididymal sperm reserves were subjected to one-way analysis of variance (ANOVA), and where significant differences existed, Duncan multiple range test was used to separate the means. ANOVA was carried out using SPSS Statistics for Windows, version 20, IBM, 2011. The values of *P* < 0.05 were considered statistically significant [21].

## Results

### General clinical observations

**Figure-1:**
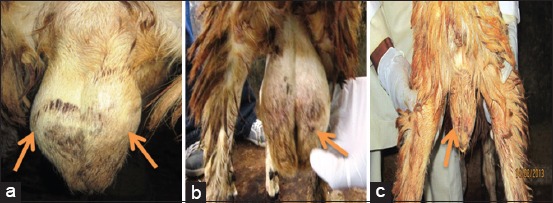
(a) Normal srotum in control ram with complete folds (arrows); (b) Scrotal edema with loss of fold (arrow) and (c) Scrotal atrophy (arrow) in *Trypanosoma brucei brucei* infected ram at day 35 and 75 post infection, respectively.

There were no clinical changes in the rams that served as control (Group IV) throughout the study period, rather, there was weight gain, increase in total plasma protein with good body condition. Observed clinical signs among the rams in the infected Groups I, II, and III were similar and include intermittent pyrexia, roughy hair coat, reduced and or selective feed intake, reduced body weight gain, pale ocular membrane with peri-orbital edema, loss of body condition, poor semen output, loss of libido, drowsiness, and death. By day 49 p.i., 1 ram (R6) in Group II (*T. evansi*) began to lose its body condition, and balance because it could not stand on its limbs. The PCV decreased severely (from mean pre-infection value of 30.0% to mean post-infection value of 13.5% by 56 days p.i.), its total plasma protein value also decreased significantly (from the pre-infection value of 6.0-3.0 by day 56 p.i.). Body weight was significantly (p<0.01) reduced from a pre-infection value of 22.95 kg to p.i. value of 16.95 kg by day 56 p.i. The ram totally lost its balance and could not ejaculate during semen collection. Animal neck became very stiff and died by the evening of day 57 p.i. Post mortem examination was done promptly. Necropsy revealed the presence of congested lungs, very pale and anemic carcass, serous atrophy of fats in the kidney, heart and intestine, severe lymphomegaly and hepatomegaly, and testicular atrophy. However, the remaining 2 rams within the group (*T. evansi*) survived the infection up to the end of the experiment as they began to recover, evidenced by gradual gain in weight, gradual increase in PCV value and the animals appeared normal by the end of the experiment (day 98 p.i) with absence of parasitemia in peripheral blood circulation. By day 75 p.i., there was severe scrotal atrophy (Figure-1c) of ram R7 in Group I (*T. brucei brucei*) as compared to those of the control Group IV that had a normal scrotum throughout the study period (Figure-1a). Before degeneration, the scrotum was swollen, soft to feel, and painful to the animal when touched (Figure-1b). As the infection progressed, another ram (R8) in Group I (*T. brucei brucei*) died by day 77 p.i. Before its death, there was decrease in weight (from pre-infection value of 23.40 kg to p.i. value of 21.44 kg at day 70 p.i.). The ram completely lost its appetite and could not stand on its limbs. The PCV decreased as low as 15% (from pre-infection value of 34% at day 0), severe decrease of scrotal circumference and the ram died overnight (day 77 p.i.). Post mortem result also revealed the presence of severely pale carcass, swollen lymph nodes, and swollen liver and serous atrophy of body fats, with severely degenerated testes (at histology). All the infected rams in Group I (*T. brucei brucei*) and Group III (mixed inoculum of *T. brucei brucei* and *T. evansi*) showed a significant (p<0.01) clinical symptoms than those rams infected in Group II (*T. evansi*) that showed mild symptoms at the chronic stage of the experiment. At few weeks (day 63 to day 98 p.i) to the end of the experiment, semen characteristics of all the infected rams, especially those of Group I and Group III, were observed to have significantly (p<0.01) deteriorated when compared to those of the uninfected control (Group IV) that had good semen characteristics. Semen volume was observed to have decreased and deteriorated in color from milky to watery appearance, as compared to creamy white to milky white in the control rams. Rams infected with either *T. brucei brucei* or mixed inoculum of the parasites (*T. brucei brucei and T. evansi*) completely lost the ability to display sexual urge (libido) when they were exposed to 2 ewes to service in the pen. However, those rams infected with *T. evansi* and those of the uninfected group that served as control were able to display a very good libido toward the ewes, but service was not allowed to take place to avoid transmission of the parasites.

### Gonadal sperm/spermatid reserve (×10^6^/g)

The live weight, scrotal circumference, testicular weight, testicular volume, and the gonadal sperm reserves of the groups at the end of 98 days p.i. period are presented in Table-2. There was a significant decrease (p<0.01) in the live weights of all the infected groups in comparison to the control Group IV. The scrotal circumferences of the infected groups decreased significantly at the end of the experiment when compared with the control Group IV (Table-2). The values of testicular length, volume, and weight of all the infected groups were found to be lower (p<0.01) than those of the control (Group IV) at the end of the experiment (Table-2). There was a drastic decrease in gonadal sperm reserve of the rams in the infected Groups I and III, which were not statistically significant difference (p>0.05) from each other but differed significantly (p<0.01) to those of Groups II and IV, respectively. The control rams had the highest gonadal sperm reserve at the end of the experiment (Table-2).

**Table-2:** Testicular length, volume, weight, and gonadal sperm reserves of Yankasa rams infected with trypanosomes, 98 days p.i (mean±SEM), n=2.

Parameter	Group I	Group II	Group III	Group IV (Control)	p value
Live weight (kg)	21.43±1.69^bc^	21.32±1.47^c^	21.70±1.63^b^	25.61±0.37^a^	<0.001**
Scrotal circumference (cm)	23.99±1.19^b^	23.53±1.56^c^	23.79±1.08^bc^	24.59±1.47^a^	<0.002**
Testis weight (g)–Right	36.79±3.43^bc^	47.51±3.94^b^	30.68±1.88^c^	90.75±3.35^a^	<0.001**
Testis weight (g)–Left	37.20±3.00^bc^	47.50±3.48^b^	30.26±1.59^c^	90.65±2.55^a^	<0.001**
Paired testis weight (g)	36.99±2.12^c^	47.51±2.12^b^	30.47±2.12^c^	90.70±2.12^a^	<0.001**
Testis length (cm)–Right	6.65±0.15^bc^	7.25±0.25^b^	6.10±0.30^c^	10.05±0.35^a^	<0.002**
Testis length (cm)–Left	6.45±0.25^bc^	7.15±0.25^b^	5.75±0.25^c^	9.85±0.35^a^	<0.002**
Paired testes length (cm)	6.55±0.20^c^	7.20±0.20^b^	5.93±0.20^c^	9.95±0.20^a^	<0.001**
Testis volume (ml)–Right	23.50±1.50^c^	40.50±0.50^b^	31.50±0.50^bc^	75.00±5.00^a^	<0.001**
Testis volume (ml)–Left	24.00±4.00^c^	38.00±0.50^b^	25.50±3.50^c^	68.50±3.50^a^	<0.002**
Gonadal sperm reserves (×10^6^/g)	40.00±2.12^c^	86.00±13.50^b^	32.00±8.82^c^	112.50±21.77^a^	<0.001**

Means with different superscripts across the same row differed significantly.

** Significant difference exists at p≤0.01 Group I (*Trypanosoma bruce brucei*); Group II (*Trypanosoma evansi*); Group III (Mixed *Trypanosoma brucei brucei* and *Trypanosoma evansi*); Group IV (uninfected control). SEM=Standard error of mean, p.i.=Post infection

### Epididymal sperm reserve (×10^6^/g)

The epididymal length, epididymal weight, and epididymal sperm reserve of experimental Yankasa rams are as presented in Table-3. Animals in the control group had the highest mean epididymal length (13.98±0.24 cm), which was statistically significant difference (p<0.01) from those of the infected Groups III (9.88±0.15 cm), II (13.14±0.17 cm), and I (11.40±0.21 cm). The same trend was observed for the epididymal weights, but those in the control group were significantly higher (p<0.01) than the infected groups (Table-3). The overall epididymal sperm reserve was highest in the control Group I (1689.50±71.71×10^6^/mL). Rams in Groups I (494.50±39.57×10^6^/mL) and III (327.50±28.40×10^6^/mL) had the lowest values of epididymal sperm reserve which were significantly lower (p<0.01) when compared with those of Groups II (776.00±44.15×10^6^/mL) and IV (1689.50±71.71×10^6^/mL) (Table-3).

**Table-3:** Epididymal length, weight, and sperm reserve of Yankasa rams infected with trypanosomes, 98 days p.i. (mean±SEM).

Parameter	Group I	Group II	Group III	Group IV (Control)	p value
Paired epididymal length (cm)	11.40±0.21^c^	13.14±0.17^b^	9.88±0.15^d^	13.98±0.24^a^	<0.001**
Weight of epididymis (g)–Right	8.43±0.19^bc^	10.02±0.75^b^	6.57±0.22^c^	20.60±0.80^a^	<0.001**
Weight of epididymis (g)–Left	8.14±0.14^c^	11.08±0.74^b^	6.76±0.05^c^	20.05±0.75^a^	<0.001**
Paired epididymal–Weight (g)	8.28±0.39^c^	10.55±0.39^b^	6.64±0.39^d^	20.33±0.39^a^	<0.001**
Epididymal sperm reserve					
Epididymal head (Caput) (×10^6^/g)	109.50±16.29^b^	116.50±4.59^b^	95.00±22.42^b^	201.50±19.97^a^	<0.001**
Epididymal body (Corpus) (×10^6^/g)	35.00±3.50^b^	72.00±4.81^b^	18.00±3.54^b^	110.25±19.32^a^	<0.001**
Epididymal tail (Cauda) (×10^6^/g)	350.50±37.73^bc^	587.50±47.10^b^	214.00±23.78^c^	1378.50±60.80^a^	<0.001**
Total	494.50±39.57^bc^	776.00±44.15^b^	327.50±28.40^c^	1689.50±71.71^a^	<0.001**

Means with different superscripts across the same row differed significantly.

** Significant difference exists at p≤0.01. Group I (*Trypanosoma brucei brucei*); Group II (*Trypanosoma evansi*); Group III (*Trypanosoma brucei brucei* and *Trypanosoma evansi* mixed); Group IV (control). SEM = Standard error of mean, p.i.=Post infection

### Gross pathology

**Figure-2:**
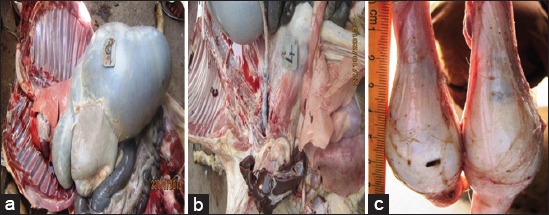
a) A normal tissue and organ architecture of control Yankasa ram with fat deposits; (b) very pale and anemic carcass (c) composition, with watery blood and severe atrophy of body fats in ram with mixed infection and testicular atrophy in infected ram.

Post mortem examination of rams that served as the uninfected control revealed no significant post mortem findings. There were normal tissues and organ architecture (Figure-2a). The rams that had mixed infections (Group III) revealed the presence of a very paled and anemic carcass composition with watery blood and severe atrophy of body fats. There was also severe testicular degeneration and splenomegaly. For *T. brucei brucei* infected rams (Group I), both the one (R8) that died and was necropsied at day 77 p.i., and the additional member of the group (R7) that was sacrificed at the end of the experiment (day 98 p.i), revealed the presence of congested and pneumonic lungs with severe hemorrhagic points, pale carcass composition (Figure-2b), serous atrophy of body fats. There was severe lymphomegaly, swollen liver (hepatomegaly), and spleen with severely degenerated testes (Figure-2c).

### Histopathology

**Figure-3:**
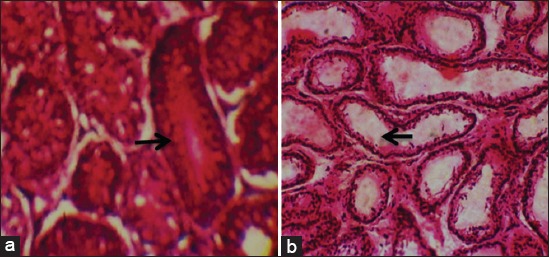
(a) Normal testes of control ram with full sperm reserve (arrow) and (b) moderately degenerated testes with severe depletion of sperm reserve (arrow) from *Trypanosoma evansi* infected ram (H and E, ×400).

**Figure-4:**
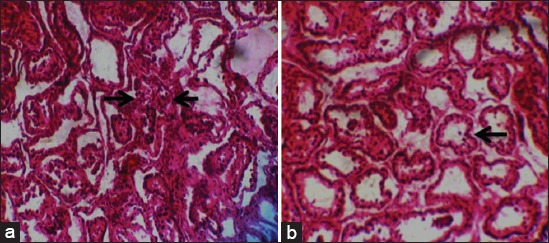
(a) Severe testicular degeneration (arrows) and depletion of sperm reserve in *Trypanosoma brucei brucei* infected rams and (b) a section of testes of mixed infection showing severe and diffused testicular degeneration with damage to Germinal and Sertoli cells (arrow) (H and E, ×400).

Histological sections of the testes of the control Yankasa rams (Group IV) showed normal tissue architecture with well define basement membrane, spermatogenic cell layers, spermatocytes with full sperm reserve in the lumen of the seminiferous tubule (Figure-3a), whereas those of the infected Yankasa rams (Groups I, II, and III) showed moderate (*T. evansi*-infected group) (Figure-3b) to severe (Mixed and *T. brucei brucei*-infected groups) testicular degenerations with reduction in number of spermatogenic cell layers, degenerated seminiferous tubules, and congested interlobular spaces with loss of tissue architecture and loss of gonadal sperm reserve in *T brucei brucei*-infected group (Figure-4a) as well as in those infected with mixed infection (Figure-4b).

## Discussion

Most of the clinical observations made during the disease may be directly attributed to the extravascular invasion by the parasites and resultant tissue lesions. Such lesions in the skin, skeletal muscles, and testicles gave rise to severe edema, which on palpation elicited painful reactions in affected animals. Similar observations were reported by Okubanjo *et al*. [5]. The swelling of the scrotum (scrotal edema) at the early stage of this experiment may be associated with inflammation process (orchitis) of the testes due to invasion by trypanosomes, thereby resulting in the increase in scrotal circumference as well as increase in body temperature. Such inflammatory processes within the testes or scrotum incited by the trypanosomes also resulted in degeneration of the testicular and scrotal tissues leading to decrease in scrotal circumference. Similarly, Okubanjo *et al*. [5] also reported variation in scrotal sizes of rams infected with *T. congolense*.

Decrease in the gonadal and epididymal sperm reserve of the infected groups as compared to the uninfected control group in this study may be attributed to the inflammatory lesions on the reproductive organs by the trypanosomes which resulted in the degeneration of the seminiferous tubules with consequence disappearance of spermatozoa, spermatids and less commonly spermatocytes. The latter results in a reduction of the mean seminiferous tubules diameter and epithelial thickness which resulted to the decreased semen volume, decreased spermatozoa concentration and motility, and increased number of abnormal and dead spermatozoa, hence leading to reduction or absence of the gonadal and epididymal sperm reserve. Impairment of the seminiferous tubule together with germ and Sertoli cells will consequently alter the process of spermatogenesis which may render the rams infertile or sterile (if untreated), and the overall effect will be a reduction or complete absence of gonadal sperm reserve. This agrees with earlier reports in boar [8], bulls [22], gazelles [4], and sheep and goat infected with *T. vivax* or *T. congolense* [5,10,23]. Testicular damage sequel to trypanosomosis in *T. brucei brucei*-infected rams and those with mixed infections compared to those of *T. evansi* infected rams, suggests that the *T. brucei brucei* strain used in this study was more virulent than the *T. evansi* strain (Sokoto isolate).

## Conclusions

Infertility caused by *T. brucei brucei*, *T. evansi*, or mixed infections in Yankasa rams is perilous. Therefore, screening for trypanosomosis in the course of investigating infertility in farm and migratory or roaming animals should be a welcome initiative, especially in tsetse-infested areas.

## Authors’ Contributions

YAW, SJO, PIR, and OOO participated in proposing and designing the experiment. YAW carried out the experiment, collected data, and drafted the manuscript. SJO, PIR, and OOO supervised the experiment. All authors read and approve the final manuscript.
